# Managing Treatment‐Emergent Immune Effector Cell‐Associated Hemophagocytic Lymphohistiocytosis‐Like Syndrome Following CAR‐T Cell Therapy: A Case‐Based Review of the use of Emapalumab

**DOI:** 10.1002/hon.70157

**Published:** 2025-11-28

**Authors:** Livia Donzelli, Veronica Zullino, Giovanni Fernando Torelli, Maria Stefania De Propris, Mario Piazzolla, Franco Ruberto, Maurizio Martelli, Alice Di Rocco

**Affiliations:** ^1^ Hematology Unit, Department of Translational and Precision Medicine Sapienza University Rome Italy; ^2^ Department of Emergency‐Acceptance, Critical Areas and Trauma Policlinico Umberto 1 Hospital Rome Italy; ^3^ Doctors with Africa CUAMM Padua Italy; ^4^ Department of Anesthesia and Intensive Care, Policlinico Umberto I Sapienza University Rome Italy; ^5^ Department of General and Specialistic Surgery Sapienza University Rome Italy

**Keywords:** brexucabtagene autoleucel, CAR‐T cell therapy, emapalumab, IEC‐HS, interferon‐γ, mantle cell lymphoma

## Abstract

Chimeric antigen receptor T (CAR‐T) cell therapies have revolutionized the treatment of hematological malignancies, achieving high response rates in patients with relapsed or refractory disease. Despite these benefits, CAR‐T cell therapies are associated with unique toxicities, including cytokine release syndrome (CRS), immune effector cell‐associated neurotoxicity syndrome (ICANS), immune cell‐associated hematotoxicity (ICAHT), and immune effector cell‐associated hemophagocytic lymphohistiocytosis‐like syndrome (IEC‐HS), which is characterized by a rare and life‐threatening hyperinflammatory response. This paper presents a case of a 56‐year‐old woman with relapsed mantle cell lymphoma (MCL) treated with the CAR‐T cell therapy, brexucabtagene autoleucel, who had subsequently developed CRS and later IEC‐HS. Initial management included tocilizumab, corticosteroids, and anakinra, followed by the compassionate use of emapalumab, an interferon‐γ blocker. To provide broader context, we conducted a literature review of CAR‐T cell‐related toxicities, focusing on IEC‐HS and its management with emapalumab. Clinical and laboratory manifestations, such as elevated ferritin levels, cytopenias, and organ dysfunction, underpin the diagnostic criteria for IEC‐HS. Vigilant monitoring and tailored therapeutic approaches are required to effectively manage toxicities associated with CAR‐T cell therapy, to maximize its benefits and minimize adverse effects. In more severe IEC‐HS cases, emapalumab may be used as an effective targeted therapy.

## Lay Summary

Chimeric antigen receptor T (CAR‐T) cell therapies are effective in killing cancer cells. These engineered therapies recognize and bind to specific proteins (or antigens) on the surface of cancer cells. However, CAR‐T cell therapies are associated with unique adverse effects, which can cause intense inflammatory responses that are unpredictable, complex and potentially life‐threatening. In this review, we present the details of a 56‐year‐old woman with mantle cell lymphoma (MCL) that had recurred despite previous treatment and who was treated with CAR‐T cell therapy (brexucabtagene autoleucel). She developed an intense inflammatory response called cytokine release syndrome as a result of treatment with brexucabtagene autoleucel, and later developed immune effector cell‐associated hemophagocytic lymphohistiocytosis‐like syndrome (IEC‐HS). Her initial treatment for IEC‐HS was with tocilizumab, corticosteroids, and anakinra, followed by the compassionate use of an interferon‐γ blocker (emapalumab). Following this treatment, her condition improved. This case suggests that emapalumab may be used as an effective targeted therapy in more severe cases of IEC‐HS. Close monitoring and individualized treatment are required to effectively manage toxicities of CAR‐T cell therapy, to maximize its benefits and minimize adverse effects. Since the effects of CAR‐T cell therapy in blood cancers can be complex, on‐going research and documentation of individual cases are essential. Emapalumab remains an effective therapeutic option for IEC‐HS, but further studies are needed to investigate how it affects CAR‐T cells.

## 1 Introduction

Chimeric antigen receptor T (CAR‐T) cell therapy involves the administration of engineered immune effector cells (IECs) expressing synthetic receptors designed to recognize antigens present on cancer cells [1]. The mechanism of action of CAR‐T cell therapy involves the activation and expansion of CAR‐T cells, targeted recognition of cancer cells expressing the antigen and the subsequent killing of these cells. The introduction of CAR‐T cell therapies has transformed the landscape of treatment for various hematological malignancies, offering high response rates for patients with relapsed or refractory disease.

To date, there are four US FDA‐approved anti‐CD19 CAR‐T cell therapies (tisagenlecleucel, axicabtagene ciloleucel, lisocabtagene maraleucel, and brexucabtagene autoleucel), and two anti‐B‐cell maturation antigen (BCMA) CAR‐T cell therapies (ciltacabtagene autoleucel and idecabtagene vicleucel) [2]. In Europe, brexucabtagene autoleucel was approved in December 2020 for the treatment of adult patients with relapsed or refractory mantle cell lymphoma (MCL) after ≥ 2 lines of systemic therapy including a Bruton's tyrosine kinase (BTK) inhibitor [3, 4]. Brexucabtagene autoleucel has been available in Italy since March 2022 and received approval based on the results of the multicenter phase 2 ZUMA‐2 trial [5, 6].

CAR‐T cell therapies are associated with unique toxicities distinct from those seen with conventional cancer treatments. Unlike traditional chemotherapy or targeted agents, CAR‐T cell‐related adverse effects stem directly from the robust activation and proliferation of engineered immune cells, which can provoke intense inflammatory responses. These responses can lead to systemic and organ‐specific toxicities that are unpredictable, complex and potentially life‐threatening [7, 8].

The severity and range of CAR‐T cell‐associated toxicities have necessitated the development of novel management protocols and grading systems to accurately assess and address these risks [9]. Toxicities such as cytokine release syndrome (CRS), immune effector cell‐associated neurotoxicity syndrome (ICANS), immune cell‐associated hematoxicity (ICAHT), and IEC‐associated hemophagocytic lymphohistiocytosis‐like syndrome (IEC‐HS) represent distinct pathological processes that require ongoing research and clinical experience to refine our understanding and treatment approaches to ensure that the benefits of CAR‐T cell therapies are fully realized [7, 8].

## 2 CAR‐T Cell Therapy‐Related Toxicities

### 2.1 CRS

CRS is the most common acute toxicity following CAR‐T cell therapy [10]. CRS occurs as a result of the rapid activation and proliferation of CAR‐T cells, which release large quantities of proinflammatory cytokines, including interleukin (IL)‐6 and interferon gamma (IFN‐γ), that further stimulate both the CAR‐T cells and the host immune system [8, 11, 12]. Characteristically, patients with CRS present initially with fever and may progress to include hypotension, capillary leakage (leading to hypoxia), and end‐organ dysfunction [9]. The incidence and severity of CRS are influenced by several factors, such as the CAR‐T product's co‐stimulatory domain, the tumor's antigen characteristics and the patient's tumor burden [8]. Diagnosis of CRS requires the exclusion of other causes of systemic inflammatory response, such as infections. Management of CRS involves supportive care and control of the inflammatory response [9]. Tocilizumab is commonly used to treat CRS as it effectively reduces inflammation without impairing CAR‐T cell efficacy [8]. According to the American Society for Transplantation and Cellular Therapy (ASTCT) consensus, patients with grade ≥ 2 CRS may require additional corticosteroids. In cases where CRS is refractory to both tocilizumab and high‐dose corticosteroids, the IL‐1 receptor antagonist anakinra is used without affecting CAR‐T cell efficacy [9].

### 2.2 ICANS

ICANS is a potentially acute development following CAR‐T cell therapy characterized by a pathological process involving the central nervous system [8, 9]. Clinically, ICANS may appear concurrently with CRS, follow the resolution of CRS, or even independently of CRS, sometimes emerging up to a month post‐infusion of CAR‐T cells [7]. Early symptoms include tremor, expressive aphasia (one of the hallmark symptoms of ICANS), impaired attention, and mild lethargy, while higher‐grade ICANS can cause refractory seizures, respiratory compromise requiring intubation, and cerebral edema. Endothelial dysfunction leading to blood‐brain barrier (BBB) disruption is believed to play a key role in the pathogenesis of ICANS [7, 8].

Several factors have been associated with an increased risk of ICANS, including a high disease burden prior to CAR‐T cell infusion, history of neurological disease, and the presence of severe CRS. Product‐specific factors, including high CAR‐T cell dose, rapid CAR‐T cell expansion kinetics, and products with CD28 co‐stimulation, also contribute to the severity of ICANS. Management of ICANS primarily involves supportive care and corticosteroids, particularly dexamethasone due to its ability to penetrate the BBB. Monitoring of neurological function using the immune effector cell‐associated encephalopathy (ICE) score is standard practice post‐CAR‐T cell infusion. In cases of concurrent CRS, tocilizumab may be considered; however, it is generally ineffective for isolated ICANS as it does not cross the BBB and it could worsen neurological symptoms due to increased free IL‐6. Anakinra has shown promise for corticosteroid‐refractory cases of ICANS and is increasingly utilized as a second‐line treatment [7, 8].

### 2.3 IEC‐HS

IEC‐HS is a severe biochemical hyperinflammatory syndrome associated with CAR‐T cell therapy (Table 1) [13], characterized by features of macrophage activation and hemophagocytic lymphohistiocytosis (HLH) [8, 13, 14]. IEC‐HS is reported in < 5% of patients, but the associated mortality rate is > 50% [8, 15].

**TABLE 1:** Definition, diagnostic criteria and clinical manifestations of IEC‐HS (adapted from Hines et al. [13]).

IEC‐HS definition	The development of a pathologic and biochemical hyperinflammatory syndrome independent from CRS and ICANS that: 1) manifests with features of macrophage activation/HLH, 2) is attributable to IEC therapy, and 3) is associated with progression or new onset of cytopenias, hyperferritinemia, coagulopathy with hypofibrinogenemia and/or transaminitis
Criteria for identification of IEC‐HS^a^	Clinical or laboratory manifestations
Most common manifestations^b^	Required: Elevated ferritin (> 2 × ULN or baseline [at time of infusion]) and/or rapidly rising (per clinical assessment)
	Onset with resolving/resolved CRS or worsening inflammatory response after initial improvement with CRS‐directed therapy^c^
	Hepatic transaminase elevation^d^ (> 5 × ULN [if baseline was normal] or > 5 x baseline if baseline was abnormal)
	Hypofibrinogenemia (< 150 mg/dL or < LLN)^e^
	Hemophagocytosis in bone marrow or other tissue^e^
	Cytopenias (new onset, worsening or refractory^f^)
Other manifestations that may be present	Lactate dehydrogenase elevations (> ULN)
	Other coagulation abnormalities (e.g., elevated PT/PTT)
	Direct hyperbilirubinaemia
	New‐onset splenomegaly
	Fevers (new^g^ or persistent)^e^
	Neurotoxicity
	Pulmonary manifestations (e.g., hypoxia, pulmonary infiltrates, pulmonary edema)
	Renal insufficiency (new onset)
	Hypertriglyceridemia (fasting level, > 265 mg/dL^e^)

Abbreviations: CRS, cytokine release syndrome; CTCAE v 5.0, Common Terminology Criteria for Adverse Events, version 5.0; HLH, hemophagocytic lymphohistiocytosis; ICANS, immune effector cell‐associated neurotoxicity syndrome; IEC, immune effector cell; IEC‐HS, immune effector cell‐associated hemophagocytic lymphohistiocytosis‐like syndrome; LLN, lower limit of normal; PT/PTT, prothrombin time/partial thromboplastin time; ULN, upper limit of normal.

^a^ Diagnosis is made only when it is not attributable to alternative etiologies, including CRS, infection and/or disease progression.

^b^ Constellation of findings, typically simultaneously (e.g., all within 72 h).

^c^ Although most cases of IEC‐HS have been seen with antecedent CRS, this may not always be the case and emerging experience will shed light on how IEC‐HS may present.

^d^ Consistent with grade 3 hepatic transaminase elevations as per CTCAE v 5.0.

^e^ As per HLH‐2004.

^f^ Generally, at least one lineage will be a grade 4 cytopenia (platelets, neutrophils, hemoglobin).

^g^ As distinguished from CRS onset or recrudescence of CRS.

The nosological entity IEC‐HS was initially defined by a commission of experts of the ASTCT in March 2023 [13]. Due to the limited number of patients and available literature and the absence of unambiguous recommendations, the commission outlined a uniform approach to managing IEC‐HS, which also led to the proposal of diagnostic criteria, including the progression or new onset of cytopenias, hyperferritinemia, coagulopathy with hypofibrinogenemia, and/or transaminitis. Key clinical and laboratory manifestations include elevated ferritin levels (> 2 × the upper limit of normal [ULN] or baseline), hepatic transaminase elevations, hypofibrinogenemia, hemophagocytosis in bone marrow or other tissues, and new or worsening cytopenias. Additional signs may include elevated LDH, other coagulation abnormalities, direct hyperbilirubinemia, new‐onset splenomegaly, persistent fevers, neurotoxicity, pulmonary manifestations (i.e., pleural effusion), renal insufficiency, and hypertriglyceridemia. While ferritin levels are markedly elevated, there is no specific cutoff in the ASTCT criteria; a rapid rise or a level above twice the ULN or baseline is considered indicative of IEC‐HS.

Diagnosis is confirmed when IEC‐HS symptoms are not attributable to alternative causes, such as CRS, infection, or disease progression [13]. Unlike CRS, which typically presents shortly after CAR‐T cell infusion, IEC‐HS tends to develop as CRS is resolving, complicating its diagnosis and management. Although the etiopathogenesis of IEC‐HS is not yet well known, it is characterized by the persistent hyperactivity of macrophages induced by the activation of CAR‐T cells mainly through the release of cytokines.

Due to the lack of clinical trials, treatment is not yet standardized and is based on the experience of treating primary and secondary HLH. There is general consensus that the first‐line drugs should be corticosteroids and anakinra [13, 16, 17]. For patients with severe or refractory IEC‐HS, additional immunosuppressive agents, such as ruxolitinib (a Janus kinase inhibitor that has shown efficacy in controlling hyperinflammatory responses by blocking signal transduction of multiple pro‐inflammatory cytokines, including IFN‐γ and IL‐6 [13, 14]), are also considered reasonable second‐line therapeutic options following corticosteroids and anakinra. Etoposide and emapalumab (a fully human immunoglobulin G1 [IgG1] monoclonal antibody against IFN‐γ, described in detail later) may also be prescribed.

Due to the limited number of patients and the paucity of literature about this rare toxicity, we report our clinical experience of the management of severe CRS/IEC‐HS with emapalumab in a case report, which illustrates the clinical course of the development of IEC‐HS and its management (including data on safety) in a patient with relapsed MCL treated with brexucabtagene autoleucel. We also present a narrative review of IEC‐HS toxicity and its management with emapalumab.

## 3 Case Report

In May 2018, a 56‐year‐old woman presented with splenomegaly (13 cm). Medical history revealed that, at the age of 35 years, she had breast cancer, which had been treated with quadrantectomy, chemotherapy, and radiotherapy. Blood tests showed a hemoglobin level of 13.8 g/dL, leukocytosis (23 × 10^3^ cells/μL), lymphocytosis (18 × 10^3^ cells/μL), and thrombocytopenia (102 × 10^3^ cells/μL). A bone marrow biopsy established the diagnosis of non‐nodal MCL with osteomedullary involvement. A “watch and wait” approach was adopted.

By December 2018, the patient's condition had progressed. Splenomegaly had increased to 17.5 cm, her lymphocyte count had increased to 91.88 × 10^3^ cells/μL and she had since developed B symptoms (night sweats, disease‐related fevers, or weight loss ≥ 10% of body weight [19]). Further genetic analysis revealed a mutation in the *TP53* gene.

Given these findings, the patient was enrolled in a clinical trial: FIL‐V‐RBAC protocol (rituximab, bendamustine, and cytarabine, followed by the BCL‐2 inhibitor venetoclax) from the Fondazione Italiana Linfomi [20]. She was treated with four cycles of R‐BAC (rituximab, bendamustine, and cytarabine), achieving a complete response (CR). This was followed by a 4‐month consolidation phase with venetoclax. However, in December 2019, venetoclax maintenance was discontinued because the patient reported poor tolerance (grade 4 neutropenia and infections), despite administration at subtherapeutic doses. In May 2021, the patient experienced her first disease relapse. Based on the findings from the MANTLE‐FIRST study [21], she began second‐line therapy with ibrutinib, a BTK inhibitor. She continued this treatment until December 2022, when a second disease relapse occurred. At this point, the patient became eligible for CAR‐T cell therapy with brexucabtagene autoleucel [4].

Prior to the CAR‐T cell infusion, the patient underwent leukapheresis and bridge therapy with pirtobrutinib, a highly selective and reversible BTK inhibitor [22]. A re‐evaluation before CAR‐T cell infusion indicated a high burden of disease. A positron emission tomography–computed tomography (PET‐CT) scan indicated a partial response, with disease localizations both above and below the diaphragm and in the spleen (measuring 17 cm). In addition, a bone marrow biopsy revealed 70%–75% of the neoplastic lymphoid component, consistent with a medullary localization of lymphoma. Therefore, the patient was considered to have an elevated tumor burden.

In April 2023, the patient was hospitalized and presented with thrombocytopenia (61 × 10^3^ cells/uL), neutropenia (0.42 × 10^3^ cells/uL), and elevated C‐reactive protein (4.82 mg/dL), indicative of an inflammatory environment. All these markers identified a high CAR‐HEMATOTOX score that has a negative prognostic value for both hematological toxicity and treatment outcomes [23, 24]. The patient received lymphodepleting chemotherapy with cyclophosphamide 500 mg/m^2^ plus fludarabine 30 mg/m^2^ from 11 to 13 April. On 18 April (Day 0), she received brexucabtagene autoleucel. Eight hours after the infusion, the patient developed CRS, which was initially classified as grade 1 [25], with a fever peak of 39°C.

**FIGURE 1:**
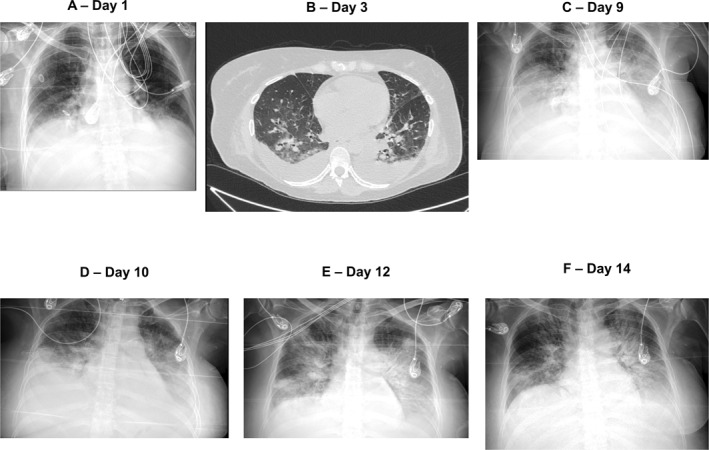
X‐rays/computed tomography scans of the patient's chest following brexucabtagene autoleucel administration on (A) day 1, (B) day 3, (C) day 9, (D) day 10, (E) day 12, and (F) day 14.

On Day 1, her condition had progressed to CRS grade 2, with fever, hypotension, and hypoxia requiring low‐flow oxygen. Consequently, she was treated with tocilizumab (8 mg/kg), anti‐IL‐6 receptor antibody, and dexamethasone 10 mg every 24 h. A chest x‐ray revealed the appearance of a pleural effusion (Figure 1a). Then, the patient was transferred to the intensive care unit (ICU).

On the second day, the patient developed CRS grade 3, characterized by fever and hypoxia requiring high‐flow oxygen. This necessitated a second dose of tocilizumab and dexamethasone 10 mg every 6 h.

**FIGURE 2:**
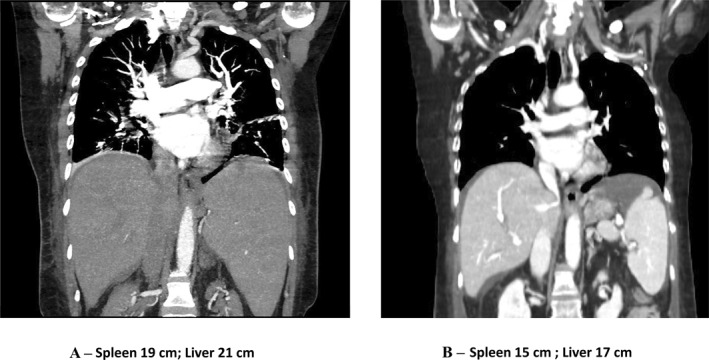
Evolution of the computed tomography scan of the patient's liver and spleen (A, B).

By the third day, her clinical condition had worsened (Figure 1b), prompting a whole‐body CT scan (Figure 2a), which revealed increased pleural effusion and splenomegaly (19 cm), as well as new findings of peritoneal effusion and hepatomegaly (21 cm). Due to persistent CRS grade 3, treatment was intensified with albumin, diuretics, and an increase in dexamethasone dose to 20 mg every 6 h.

On the fourth day, there was no improvement. Therefore, it was decided to add anakinra subcutaneously every 6 h and methylprednisolone 1 g/day.

**FIGURE 3:**
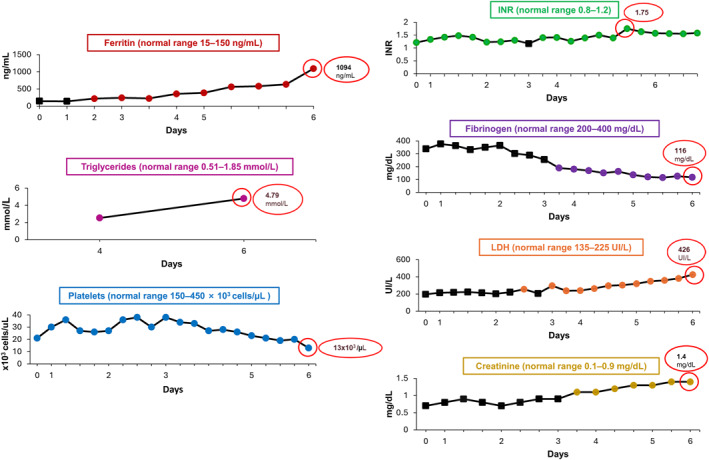
Laboratory and hematological parameters during days 0–6 following brexucabtagene autoleucel administration. Black squares indicate readings in the normal range. INR, international normalized ratio; LDH, lactate dehydrogenase.

Despite these interventions, on the sixth day, the patient's respiratory condition deteriorated further, necessitating the use of high‐flow positive pressure nasal cannulas, indicating grade 4 CRS resistant to high‐dose corticosteroid therapy. Laboratory data following CAR‐T cell infusion showed a progressive increase in ferritin (1094 ng/mL) and triglycerides (4.79 mmol/L), thrombocytopenia unresponsive to transfusion therapy (13 × 10^3^ cells/μL), coagulopathy (International normalized ratio, INR: 1.75) with hypofibrinogenemia (116 mg/dL) despite plasma therapy, and increased levels of lactate dehydrogenase (LDH: 426 IU/L) and creatinine (1.4 mg/dL; Figure 3). These findings, combined with worsening splenomegaly, persistent fever and pulmonary symptoms, raised the suspicion of IEC‐HS [13]. Due to the deteriorating clinical condition of the patient and based on recent case reports and studies [26, 27, 28], the compassionate use of emapalumab was requested and started on Day 6. Emapalumab was administered at a dose of 4 mg/kg, which could have been readministered every 3 days until clinical improvement was observed. In addition, the patient continued therapy with anakinra and methylprednisolone 1 g/day.

**FIGURE 4:**
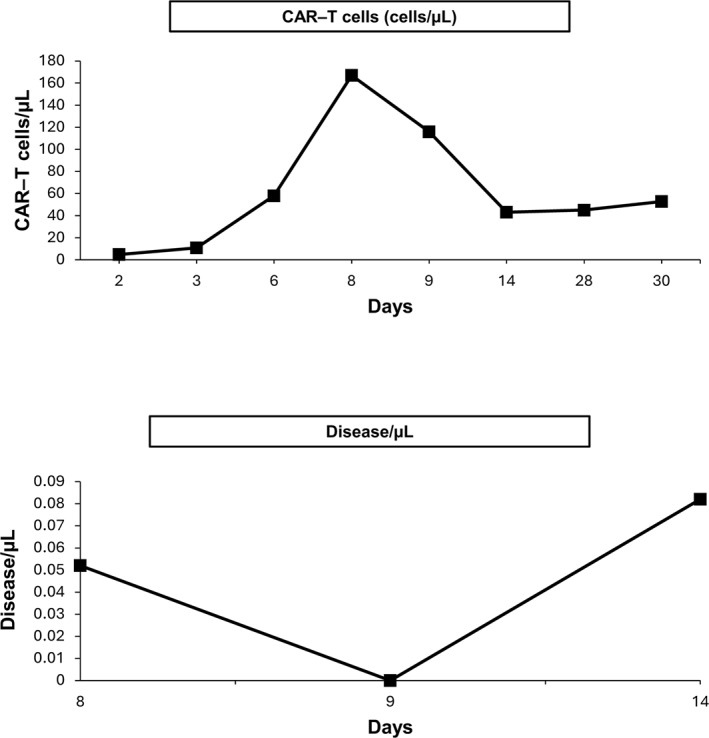
Expansion curve of CAR‐T cells and disease monitoring assessed by flow cytometry.

On the ninth day, the patient experienced an increase of pleural effusion, leading to an evacuative thoracentesis (Figure 1c). Flow cytometry of the pleural fluid revealed 80% CAR‐T cells, with no evidence of disease; the flow cytometry trend of CAR‐T cells and CD19‐negative cells showed that the peak levels of CAR‐T cells corresponded to the absence of disease, but coincided with the maximum toxicity (Figure 4). Consequently, methylprednisolone 2 g was administered on Day 9.

**FIGURE 5:**
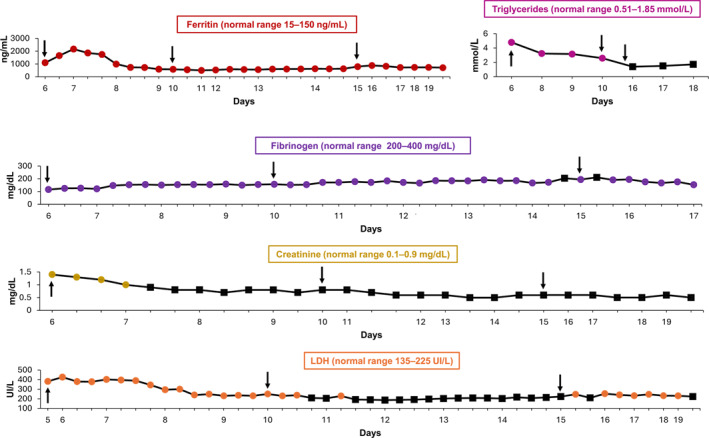
Laboratory and hematological parameters during days 6–18 following emapalumab administration. Black squares indicate readings in the normal range. Arrows indicate the days that emapalumab was administered. INR, international normalized ratio; LDH, lactate dehydrogenase.

The second and third doses of emapalumab were administered on the 10^th^ and 15^th^ days, respectively. From the 11^th^ day, there was progressive improvement in pulmonary condition (Figure 1d,e,f). In addition, laboratory parameters began to normalize, with reductions in ferritin, triglycerides, LDH and creatinine, and an increase in fibrinogen (Figure 5).

On the 18^th^ day, the patient was discharged from the ICU and transferred to the hematology department and within 5 days, oxygen therapy was discontinued. A whole‐body CT scan showed a reduction in lymphadenopathy and hepatosplenomegaly (Figure 2b). The bone marrow biopsy remained positive. All these findings indicated a partial response to CAR‐T cell therapy. On 25 May, after almost 2 months of hospitalization, the patient was discharged. After 1 month, the patient experienced central nervous system disease progression, accompanied by a progressive clinical deterioration, and unfortunately died in November 2023.

## 4 Mechanism of IEC‐HS

The underlying mechanisms of IEC‐HS involve sustained T cell activation and a hyperinflammatory feedback loop triggered by CAR‐T engagement with tumor antigens [7, 8]. This results in macrophage activation and widespread cytokine release, with cytokines such as IL‐1β, IL‐6, IFN‐γ, and tumor necrosis factor (TNF)‐α playing central roles. Unlike CRS, where IL‐6 predominates, IEC‐HS involves IL‐1 as a primary driver of inflammation and IFN‐γ is thought to play a significant role in macrophage activation. Other immune parameters, such as elevated IL‐18 and sIL‐2R, as well as a high ferritin, overlap with severe CRS and IEC‐HS.

There are multiple risk factors for IEC‐HS, including baseline disease burden, CAR‐T cell proliferation dynamics, and patient‐specific characteristics, such as baseline inflammation and immune suppression. The specific CAR‐T cell construct, including factors such as target antigen and co‐stimulatory domains, may also contribute to IEC‐HS incidence and severity. Studies suggest that lower pre‐infusion natural killer (NK) cell levels and higher ratios of T cells to NK cells in the bone marrow may predict the development of IEC‐HS in some patients [7].

Management of IEC‐HS requires a tailored therapeutic approach to mitigate severe toxicities without compromising the anti‐tumor efficacy of CAR‐T cells. With corticosteroids and anakinra established as the first‐treatment of IEC‐HS [13], experts recommend ruxolitinib [18] as a second‐line agent and, in severe cases, low doses of etoposide or emapalumab (recently approved for primary HLH) [17].

## 5 Role of Emapalumab in IEC‐HS

Emapalumab, a fully human monoclonal antibody directed against IFN‐γ, was initially approved for primary HLH and is now being explored for secondary HLH‐like conditions such as IEC‐HS [29, 30]. By neutralizing IFN‐γ, emapalumab dampens the hyperinflammatory state, thereby mitigating tissue injury and improving clinical outcomes. A few case series and preliminary reports suggest that emapalumab is effective in reversing disease progression in patients with corticosteroid and anakinra refractory IEC‐HS and is generally well tolerated [26, 27, 28]. However, controlled clinical trials are lacking, and the role of emapalumab in the IEC‐HS treatment algorithm remains to be formally established.

Locatelli and colleagues evaluated emapalumab in children with primary HLH and found that emapalumab may be employed as targeted therapy in more severe, refractory cases [30]. Emapalumab binds both free and receptor‐bound IFN‐γ, neutralizing its activity by preventing receptor dimerization and subsequent signal transduction, effectively dampening the IFN‐γ‐driven hyperinflammatory response characteristic of IEC‐HS. Emapalumab has shown promising efficacy in controlling severe inflammation, with a rapid reduction in CXCL9 levels (a biomarker of IFN‐γ activity) in primary HLH. This reduction was associated with improved clinical outcomes, thus making emapalumab a relevant therapeutic option in IEC‐HS. The safety profile of emapalumab has been favorable, with no evidence of significant hematological, hepatic, or renal toxicity attributable to the drug. Adverse events, particularly infections, can be a concern due to the immunosuppressed state of patients. However, in the study by Locatelli et al., most of the infections had resolved and there were no myelosuppressive effects directly attributed to emapalumab.

Primarily based on the comparison between this study and that of Böhm et al. [31] on the use of etoposide in primary hemophagocytic lymphohistiocytosis, the best practice recommendations issued in April 2025 by the EBMT Practice Harmonization and Guidelines Committee [17] (addressing the management of critical post–CAR T‐cell complications, including HLH) recommended the systematic use of low‐dose etoposide as a third‐line salvage treatment for refractory cases.

However, the review of recent literature allows us to highlight two case reports (similar to our own) illustrating the successful use of emapalumab in severe HLH/macrophage activation syndrome secondary to CAR‐T cell therapy in two patients with relapsed/refractory B‐cell acute lymphoblastic leukemia [26, 27]. The achievement of complete response indicated that emapalumab did not have deleterious effects on the efficacy of CAR‐T cell therapy. In addition, in the most recently published case report [27], the treatment included high‐dose corticosteroids, anakinra, siltuximab, and ruxolitinib. Despite these ongoing therapies, emapalumab was necessary for complete resolution.

## 6 Discussion

This case report illustrates the successful use of emapalumab in a patient who had developed IEC‐HS secondary to CAR‐T cell therapy. The risk factors that led to severe toxicity were high disease burden, leukemization, CAR‐T cells with CD28 costimulatory domain, and the baseline inflammatory characteristics of the patient (thrombocytopenia, neutropenia, elevated C‐reactive protein, and high CAR‐HEMATOTOX score). Because the patient was refractory to corticosteroids, tocilizumab, and anakinra, we decided to administer emapalumab. Therefore, this case supports the use of an anti‐IFN‐γ agent to manage HLH. The patient initially achieved a partial response, followed by disease progression. Evidence regarding the impact of emapalumab on the efficacy of CAR‐T cell immunotherapy remains limited. Preclinical studies demonstrate that IFN‐γ inhibition does not impair CAR‐T cell function [28], and several case reports suggest that emapalumab does not hinder antitumor activity. Nevertheless, prospective studies are warranted to systematically evaluate both short‐ and long‐term effects of emapalumab on CAR‐T cell efficacy and persistence, given the key modulatory role of IFN‐γ.

IEC‐HS is a rare but potentially fatal hyperinflammatory complication that may arise following immune effector cell therapies, particularly CAR‐T cell therapy. It is characterized by clinical and laboratory features overlapping with classical HLH and macrophage activation syndrome [8, 15]. IEC‐HS typically develops during the resolution phase of CRS, especially in patients who experienced high‐grade CRS requiring immunomodulatory interventions such as tocilizumab and high‐dose corticosteroids. This temporal association underscores the evolving immunopathology in the post‐CRS setting, marked by sustained macrophage activation and dysregulated cytokine production [8].

Timely suspicion and identification of IEC‐HS signs and symptoms are imperative, as this facilitates more rapid laboratory data evaluation and reduces diagnostic delays, which are associated with clinical decline and increased mortality. Hallmark features of IEC‐HS include persistent or recurrent fever, elevated ferritin levels, progressive or new‐onset cytopenias, organ dysfunction (hepatic, renal, or pulmonary), and coagulopathy, often manifesting as hypofibrinogenemia [13]. These manifestations can mimic or coexist with CRS or sepsis, complicating the diagnostic process. Therefore, a high index of suspicion is required, particularly in patients with prolonged inflammatory symptoms or incomplete resolution of CRS despite standard treatment.

Since IEC‐HS is a potentially fatal toxicity associated with CAR‐T cell therapy, early recognition with reliable diagnostic criteria and initiating prompt treatment specific to IEC‐HS is imperative for improving patient outcomes. Although evidence is scant, the success of therapy after emapalumab is supported by a few case reports and, thus, emapalumab should be investigated further. In cases where IEC‐HS persists despite emapalumab treatment, additional therapies, including low‐dose etoposide for selective T cell depletion, may be considered.

From a pathophysiological perspective, IFN‐γ plays a central role in the development of IEC‐HS [8]. As a potent activator of macrophages and dendritic cells, IFN‐γ contributes to a self‐amplifying inflammatory cascade that drives hemophagocytic activity and systemic immune dysregulation [15]. In this context, targeting IFN‐γ has emerged as a rational therapeutic strategy, particularly in cases refractory to high‐dose corticosteroids and anakinra. Further research is warranted to elucidate optimal diagnostic criteria, timing of interventions, and long‐term outcomes associated with IFN‐γ blockade in this setting. Additionally, defining the risk factors for IEC‐HS development post‐CAR‐T cell therapy could enable preemptive strategies in high‐risk individuals.

IEC‐HS represents a critical and under‐recognized toxicity of CAR‐T cell therapy, requiring prompt identification and targeted immunosuppression. Emapalumab offers a promising therapeutic option in severe and corticosteroid‐refractory cases. Over the next few years, advancing our understanding of IEC‐HS pathogenesis and establishing the role of human IgG1 monoclonal antibodies targeting IFN‐γ will be essential for standardizing targeted therapeutic strategies, improving patient outcomes, and enhancing the safety profile of CAR‐T cell therapies. As a result, the current empiric, stepwise escalation of immunosuppression may be replaced by a more targeted and personalized approach. However, challenges such as regulatory approval, access to novel therapies, and the need for multicenter collaboration to address rare complications like IEC‐HS must be overcome to fully realize this evolving treatment paradigm.

## 7 Conclusion

Since IEC‐HS therapy is a complication that has been recently defined and is characterized by high mortality, further studies are necessary to better understand its etiopathogenesis and establish the most appropriate management strategy. IEC‐HS represents a severe hyperinflammatory response that can develop as CRS resolves. The diagnostic criteria for IEC‐HS involve a combination of clinical and laboratory manifestations, including elevated ferritin levels, cytopenias, and organ dysfunction. The case report described herein highlights the need for vigilant monitoring and tailored therapeutic approaches to manage toxicities effectively, ensuring that the benefits of CAR‐T cell therapy are maximized, while adverse effects are minimized. The case report also illustrates the complex clinical course following CAR‐T cell therapy, underscoring the importance of real‐world data in refining management strategies and improving patient outcomes. As the understanding of IEC‐HS continues to evolve, further research will be essential to optimize treatment protocols and enhance the safety profile of CAR‐T cell therapies. Emapalumab remains a valid therapeutic option, but prospective studies are needed to investigate its efficacy and safety in terms of its ensuing impact on CAR‐T cells.

## Author Contributions

All authors contributed to the conception of the manuscript under the coordination of A.D.R., A.D.R. and L.D. reviewed the literature and critically reviewed the manuscript. All authors read and approved the final version of the manuscript.

## Funding

This work and the Open Access were supported by Sobi.

## Consent

The next of kin of the patient in this case report provided written informed consent to publish their data.

## Conflicts of Interest

M.M. has received speaker honoraria from Roche, Gilead, Recordati, Incyte, Janssen‐Cilag, and BeiGene; and fees for advisory board participation from Roche, Gilead, Novartis, Takeda, Incyte, Recordati, Janssen‐Cilag, BeiGene, AstraZeneca, and BMS. A.D.R. has received speaker honoraria from Roche, Gilead, Janssen, and AbbVie; grants from Takeda and Gilead; and consulting fees from Roche, Takeda, Incyte, Gilead, Novartis, Eli Lilly, and AstraZeneca. All other authors (L.D., V.Z., G.F.T., M.S.D.P., M.P., and F.R.) have no conflicts of interest to declare.

## Data Availability

Data sharing is not applicable to this article as no new data were created or analyzed in this case‐based review.

## References

[1] E. Bourbon , H. Ghesquières , and E. Bachy , “CAR‐T Cells, From Principle to Clinical Applications,” Bull Cancer 108, no. 10s (2021): S4–s17, 10.1016/j.bulcan.2021.02.017. 34920806

[2] M. Z. Ong , S. A. Kimberly , W. H. Lee , et al., “FDA‐Approved CAR T‐Cell Therapy: A Decade of Progress and Challenges,” Current Pharmaceutical Biotechnology 25, no. 11 (2024): 1377–1393, 10.2174/0113892010257212231001082741. 39034731

[3] Z. Huang , V. P. Chavda , R. Bezbaruah , H. Dhamne , D. H. Yang , and H. B. Zhao , “CAR T‐Cell Therapy for the Management of Mantle Cell Lymphoma,” Molecular Cancer 22, no. 1 (2023): 67, 10.1186/s12943-023-01755-5. 37004047 PMC10064560

[4] European Medicines Agency , “Tecartus: EPAR ‐ all Authorised Presentations,”2021, Accessed 22 July, 2024, https://www.ema.europa.eu/en/medicines/human/EPAR/tecartus.

[5] M. Wang , J. Munoz , A. Goy , et al., “Three‐Year Follow‐Up of KTE‐X19 in Patients With Relapsed/Refractory Mantle Cell Lymphoma, Including High‐Risk Subgroups, in the ZUMA‐2 Study,” Journal of Clinical Oncology 41, no. 3 (2023): 555–567, 10.1200/jco.21.02370. 35658525 PMC9870225

[6] M. Wang , J. Munoz , A. Goy , et al., “KTE‐X19 CAR T‐Cell Therapy in Relapsed or Refractory Mantle‐Cell Lymphoma,” New England Journal of Medicine 382, no. 14 (2020): 1331–1342, 10.1056/nejmoa1914347. 32242358 PMC7731441

[7] C. J. Ferreri and M. Bhutani , “Mechanisms and Management of CAR T Toxicity,” Frontiers Oncology 14 (2024): 1396490, 10.3389/fonc.2024.1396490. PMC11148294 38835382

[8] A. D. Hughes , D. T. Teachey , and C. Diorio , “Riding the Storm: Managing Cytokine‐Related Toxicities in CAR‐T Cell Therapy,” Seminars in Immunopathology 46, no. 3–4 (2024): 5, 10.1007/s00281-024-01013-w. 39012374 PMC11252192

[9] D. W. Lee , B. D. Santomasso , F. L. Locke , et al., “ASTCT Consensus Grading for Cytokine Release Syndrome and Neurologic Toxicity Associated With Immune Effector Cells,” Biology of Blood and Marrow Transplantation 25, no. 4 (2019): 625–638, 10.1016/j.bbmt.2018.12.758. 30592986 PMC12180426

[10] D. C. Fajgenbaum and C. H. June , “Cytokine Storm,” New England Journal of Medicine 383, no. 23 (2020): 2255–2273, 10.1056/nejmra2026131. 33264547 PMC7727315

[11] D. T. Teachey , S. F. Lacey , P. A. Shaw , et al., “Identification of Predictive Biomarkers for Cytokine Release Syndrome After Chimeric Antigen Receptor T‐Cell Therapy for Acute Lymphoblastic Leukemia,” Cancer Discovery 6, no. 6 (2016): 664–679, 10.1158/2159-8290.cd-16-0040. 27076371 PMC5448406

[12] C. Diorio , R. Shraim , R. Myers , et al., “Comprehensive Serum Proteome Profiling of Cytokine Release Syndrome and Immune Effector Cell‐Associated Neuro‐Toxicity Syndrome Patients With B‐cell ALL Receiving CAR T19,” Clinical Cancer Research 28, no. 17 (2022): 3804–3813, 10.1158/1078-0432.ccr-22-0822. 35705524 PMC9444956

[13] M. R. Hines , T. E. Knight , K. O. McNerney , et al., “Immune Effector Cell‐Associated Hemophagocytic Lymphohistiocytosis‐Like Syndrome,” Transplant Cell Ther 29, no. 7 (2023): 438, 10.1016/j.jtct.2023.03.006. PMC10330221 36906275

[14] J. I. Henter , A. Horne , M. Aricó , et al., “HLH‐2004: Diagnostic and Therapeutic Guidelines for Hemophagocytic Lymphohistiocytosis,” Pediatric Blood and Cancer 48, no. 2 (2007): 124–131, 10.1002/pbc.21039. 16937360

[15] T. Fugere , A. Baltz , A. Mukherjee , et al., “Immune Effector Cell‐Associated HLH‐like Syndrome: A Review of the Literature of an Increasingly Recognized Entity,” Cancers 15, no. 21 (2023): 5149, 10.3390/cancers15215149. 37958323 PMC10647774

[16] J. C. Lee , W. T. Johnson , M. Hines , and N. N. Shah , “Immune Effector Cell‐Associated Hemophagocytic Lymphohistiocytosis‐Like Syndrome (IEC‐HS),” Hematology‐Oncology Clinics of North America 39, no. 3 (2025): 617–643, 10.1016/j.hoc.2025.02.005. 40158936 PMC12365782

[17] G. Ortí , G. Dachy , C. E. Graham , et al., “Less Frequent Complications Following CAR T‐Cell Therapies: Hemophagocytic Lymphohistiocytosis, Graft‐Versus‐Host Disease, Thrombotic Microangiopathy, Coagulation Disorders and Secondary Malignancies: Best Practice Recommendations From the EBMT Practice Harmonization and Guidelines Committee,” Bone Marrow Transplantation 60, no. 6 (June 2025): 751–758, 10.1038/s41409-025-02567-5. 40205032

[18] K. O. McNerney , A. M. DiNofia , D. T. Teachey , S. A. Grupp , and S. L. Maude , “Potential Role of IFNγ Inhibition in Refractory Cytokine Release Syndrome Associated With CAR T‐Cell Therapy,” Blood Cancer Discovery 3, no. 2 (2022): 90–94, 10.1158/2643-3230.bcd-21-0203. 35015687 PMC9245357

[19] P. Jain and M. Wang , “Mantle Cell Lymphoma: 2019 Update on the Diagnosis, Pathogenesis, Prognostication, and Management,” American Journal of Hematology 94, no. 6 (2019): 710–725, 10.1002/ajh.25487. 30963600

[20] C. Visco , V. Tabanelli , M. V. Sacchi , et al., “Rituximab, Bendamustine and Cytarabine Followed by Venetoclax (V‐RBAC) in High‐Risk Older Patients With Mantle Cell Lymphoma: A Phase 2 Study by the Fondazione Italiana Linfomi (FIL),” supplement, Blood 142, no. S1 (2023): 737, 10.1182/blood-2023-179169.

[21] C. Visco , A. Di Rocco , A. Evangelista , et al., “Outcomes in First Relapsed‐Refractory Younger Patients With Mantle Cell Lymphoma: Results From the MANTLE‐FIRST Study,” Leukemia 35, no. 3 (2021): 787–795, 10.1038/s41375-020-01013-3. 32782382

[22] A. R. Mato , N. N. Shah , W. Jurczak , et al., “Pirtobrutinib in Relapsed or Refractory B‐Cell Malignancies (BRUIN): A Phase 1/2 Study,” Lancet 397, no. 10277 (2021): 892–901, 10.1016/s0140-6736(21)00224-5. 33676628 PMC11758240

[23] K. Rejeski , M. Subklewe , M. Aljurf , et al., “Immune Effector cell‐Associated Hematotoxicity: EHA/EBMT Consensus Grading and Best Practice Recommendations,” Blood 142, no. 10 (2023): 865–877, 10.1182/blood.2023020578. 37300386

[24] F. Stella , A. Chiappella , B. Casadei , et al., “A Multicenter Real‐Life Prospective Study of Axicabtagene Ciloleucel Versus Tisagenlecleucel Toxicity and Outcomes in Large B‐cell Lymphomas,” Blood Cancer Discovery 5, no. 5 (2024): 318–330, 10.1158/2643-3230.bcd-24-0052. 38953781 PMC11369587

[25] P. J. Hayden , C. Roddie , P. Bader , et al., “Management of Adults and Children Receiving CAR T‐Cell Therapy: 2021 Best Practice Recommendations of the European Society for Blood and Marrow Transplantation (EBMT) and the Joint Accreditation Committee of ISCT and EBMT (JACIE) and the European Haematology Association (EHA),” Annals of Oncology 33, no. 3 (2022): 259–275, 10.1016/j.annonc.2021.12.003. 34923107

[26] M. Rainone , D. Ngo , J. H. Baird , et al., “Interferon‐γ Blockade in CAR T‐Cell Therapy‐associated Macrophage Activation Syndrome/hemophagocytic Lymphohistiocytosis,” Blood Advances 7, no. 4 (2023): 533–536, 10.1182/bloodadvances.2022008256. 35917457 PMC9979758

[27] B. Manghisi , G. Cotilli , M. Fedele , et al., “Case Report: Successful Use of Emapalumab in Adult B‐Cell Acute Lymphoblastic Leukemia Experiencing Severe Neurotoxicity and Hemophagocytic Lymphohistiocytosis‐like Features After CAR‐T Cell Therapy,” Frontiers in Immunology 16 (April 2025): 1563736, 10.3389/fimmu.2025.1563736. 40255392 PMC12006129

[28] S. Manni , F. Del Bufalo , P. Merli , et al., “Neutralizing IFNγ Improves Safety Without Compromising Efficacy of CAR‐T Cell Therapy in B‐cell Malignancies,” Nature Communications 14, no. 1 (2023): 3423, 10.1038/s41467-023-38723-y. PMC10256701 37296093

[29] P. Merli , M. Algeri , S. Gaspari , and F. Locatelli , “Novel Therapeutic Approaches to Familial HLH (Emapalumab in FHL),” Frontiers in Immunology 11 (2020): 608492, 10.3389/fimmu.2020.608492. 33424859 PMC7793976

[30] F. Locatelli , M. B. Jordan , C. Allen , et al., “Emapalumab in Children With Primary Hemophagocytic Lymphohistiocytosis,” New England Journal of Medicine 382, no. 19 (2020): 1811–1822, 10.1056/nejmoa1911326. 32374962

[31] S. Böhm , K. Wustrau , J. Pachlopnik Schmid , et al., “Survival in Primary Hemophagocytic Lymphohistiocytosis, 2016 to 2021: Etoposide is Better Than Its Reputation,” Blood 143, no. 10 (2024): 872–881, 10.1182/blood.2023022281. 37992218

